# Calcium Signaling and Cardiac Adaptation to Stress: Focus on Pregnancy and Diabetes

**DOI:** 10.3390/biom15101421

**Published:** 2025-10-07

**Authors:** Sathya Velmurugan, Sanda Despa

**Affiliations:** 1 Department of Internal Medicine, Division of Endocrinology, Diabetes and Metabolism, University of Kentucky, Lexington, KY 40536, USA; sathya.velmurugan@uky.edu; 2 Department of Pharmacology and Nutritional Sciences, University of Kentucky, Lexington, KY 40536, USA

**Keywords:** Ca^2+^ signaling, pregnancy, diabetes, cardiac remodeling

## Abstract

Calcium (Ca^2+^) signaling regulates a wide range of processes in the heart, from contractility and excitability to energy supply and cell growth. Consequently, Ca^2+^ signaling plays a critical role in cardiac adaptation to both physiological and pathophysiological stress. This review examines the role of Ca^2+^ signaling in the heart’s physiological adaptation to pregnancy and its pathological maladaptation in diabetes. We focus on Ca^2+^-dependent mechanisms involved in hypertrophy, energy imbalance, and electrical remodeling in these two conditions, highlighting shared signaling pathways, functional outcomes, and key knowledge gaps. A deeper understanding of these mechanisms could reveal novel therapeutic targets to improve cardiac health in pregnancy and diabetes.

## 1. Introduction

Cardiac Ca^2+^ signaling is most often associated with its role in triggering contraction. In this process, membrane depolarization during an action potential promotes Ca^2+^ entry into cardiac myocytes via voltage-gated L-type Ca^2+^ channels [1,2]. This Ca^2+^ influx causes a localized rise in intracellular Ca^2+^ concentration ([Ca^2+^]_i_), which induces Ca^2+^ release from the sarcoplasmic reticulum (SR) via activation of ryanodine receptors (RyRs). The resulting increase in [Ca^2+^]_i_ facilitates the binding of Ca^2+^ to the myofilaments, initiating contraction. Ca^2+^ is subsequently cleared from the cytosol by reuptake into the SR via the sarco/endoplasmic reticulum Ca^2+^ ATPase (SERCA) pump and extrusion from the cell through the sarcolemmal Na^+^/Ca^2+^ exchanger (NCX), leading to relaxation [1,2].

In addition to its essential role in driving contraction, Ca^2+^ signaling regulates numerous other processes in the heart, including gene expression, electrical activity, and cellular metabolism. Ca^2+^-dependent activation of calcineurin [3,4] and Ca^2+^/calmodulin-dependent protein kinase II (CaMKII) [5,6] promotes the transcription of genes involved in myocyte growth, contributing to cardiac hypertrophy. CaMKII also regulates the function of several ion channels and transporters, including RyRs and voltage-gated Na^+^, Ca^2+^, and K^+^ channels (extensively reviewed in [7,8,9,10,11]). Through these actions, CaMKII directly affects the overall electrical activity of the heart and arrhythmogenesis. Within mitochondria, Ca^2+^ activates pyruvate dehydrogenase, α-ketoglutarate dehydrogenase, and isocitrate dehydrogenase, key enzymes of the Krebs cycle. As a result, mitochondrial Ca^2+^ plays a central role in controlling both energy metabolism and redox homeostasis [12,13,14,15].

**Figure 1:**
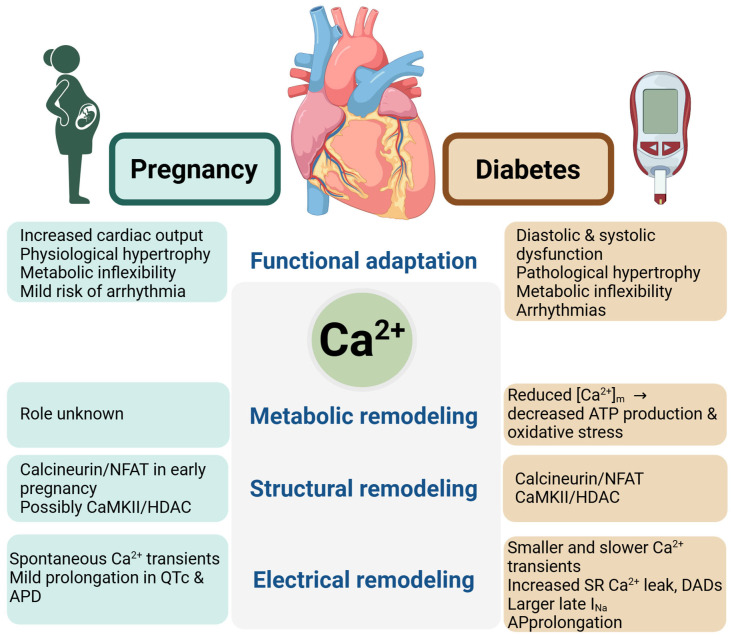
Role of Ca^2+^ signaling in cardiac physiological adaptation to pregnancy and pathological maladaptation in diabetes. Ca^2+^ signaling contributes to metabolic, structural, and electrical remodeling of the heart under both conditions. Key pathways and molecules involved are indicated. During pregnancy, Ca^2+^-driven remodeling supports physiological adaptation and is largely reversible, whereas in diabetes, similar remodeling mechanisms become maladaptive and irreversible, resulting in progressive functional impairment. Created in https://BioRender.com (accessed on 29 August 2025).

Because of its involvement in such diverse cellular functions, Ca^2+^ signaling is a key mediator of the heart’s response to both physiological and pathophysiological stress. This review focuses on the contribution of Ca^2+^ signaling to the physiological adaptation of the heart to pregnancy and its maladaptive remodeling in the context of diabetes (Figure 1).

## 2. Cardiac Adaptation to Pregnancy

Pregnancy induces profound hemodynamic changes, as the blood volume increases up to ~50% and systemic vascular resistance drops by ~35–40% compared to pre-pregnancy levels [16]. The decrease in vascular resistance is primarily mediated by vasodilation in response to elevated circulating levels of estrogen, progesterone, and relaxin [17]. These adaptations promote cardiac hypertrophy [16,18,19,20,21] and a corresponding increase in cardiac output generally starting in the second trimester [16,17,19,22]. In the third trimester, an acceleration of the heart rate further contributes to sustaining the elevated cardiac output [17]. The corrected QT interval (QTc) is significantly longer, and QT dispersion is greater in pregnant women compared to non-pregnant women of similar age [23,24,25,26]. Despite the increase in QTc duration, the overall risk of arrhythmias during pregnancy remains low. However, an upward trend in the incidence of pregnancy-related arrhythmias, particularly atrial fibrillation, has been observed over the past 20 years [26,27].

Pregnancy-induced cardiac hypertrophy is a physiological process, characterized by mild, proportional increases in left-ventricular diameter and wall thickness (eccentric hypertrophy) [16,18,19,20,21]. Typical to physiological hypertrophy, the heart growth during pregnancy occurs in the absence of fibrosis [21,28,29] or upregulation of fetal hypertrophic genes such as β-myosin heavy chain, atrial natriuretic peptide, and brain natriuretic peptide [20,29]. This change in heart size and structure is reversible within 2–4 months following a normal, healthy pregnancy [19,30]. However, adverse pregnancy outcomes such as gestational diabetes are associated with long-term cardiac hypertrophy and dysfunction [31,32,33,34,35].

Under normal physiological conditions, when oxygen and fuel are abundant, the myocardium primarily relies on fatty acids, which provide about 70% of its energy requirements, while glucose contributes the remaining ~30% [36,37]. During pregnancy, the increased workload imposes a substantial metabolic stress on the heart. Pregnancy also induces systemic insulin resistance, a physiological adaptation that helps prioritize the energetic demands of the fetus and its preference for glucose [17,37]. As a result, myocardial glucose utilization declines during pregnancy, primarily due to the upregulation of pyruvate dehydrogenase kinase-4 [29], and the heart relies even more heavily on fatty acid oxidation for generating ATP [37]. Moreover, there is an increased utilization of ketone bodies as an alternative fuel under certain conditions [29,36,37,38]. Despite this metabolic shift, the oxygen consumption rate of mitochondria isolated from hearts of pregnant mice is comparable to that of non-pregnant controls [38,39]. However, myocardial expression of peroxisome proliferator-activated receptor gamma coactivator 1α (PGC-1α), the “master regulator” of mitochondrial biogenesis, is elevated during pregnancy [40]. These findings suggest that the heart meets its increased energy demands during pregnancy through increased mitochondrial biogenesis rather than by enhancing the function of individual mitochondria.

Cardiac adaptation to the increased workload and metabolic demands of pregnancy is likely influenced by factors such as maternal age and overweight/obesity. In female rats, aging has been linked to impaired cardiac mitochondrial respiration, and the effect is further worsened by elevated Ca^2+^ levels [41]. Given the rising trends in maternal age and the prevalence of overweight and obesity, further research is needed to understand how these factors affect cardiac adaptation during pregnancy and recovery in the postpartum period.

## 3. Cardiac Remodeling in Diabetes

Diabetes significantly increases the risk for cardiovascular disease, including coronary artery disease, myocardial infarction, and heart failure (reviewed in [42,43]). Moreover, heart failure patients with diabetes experience higher rates of mortality and hospitalization compared to those with heart failure alone [44]. Major structural, electrical, and functional remodeling of the heart can occur even in the absence of vascular complications [45,46,47,48,49]. Diastolic dysfunction [42,46,47,48,50] and cardiac hypertrophy [45,51,52,53] are early features of diabetic heart disease, often emerging during the prediabetic, insulin-resistant phase. In many cases, this remodeling advances to systolic dysfunction following the onset of diabetes [45,54]. Notably, the risk of heart failure is heightened even in individuals who do not progress to overt diabetes [55,56].

Diabetes-induced structural remodeling of the heart typically begins as concentric hypertrophy and may evolve into eccentric hypertrophy with left-ventricular dilation in more advanced stages of the disease [45,52,57]. Unlike the physiological hypertrophy observed during pregnancy, the increase in cardiac mass in diabetes is accompanied by fibrosis [42,58,59,60,61,62] and re-expression of fetal hypertrophic genes [63].

Metabolic alterations are a central contributor to diabetes-associated heart dysfunction. The diabetic heart exhibits reduced metabolic flexibility, with increased reliance on fatty acid oxidation and decreased glucose uptake, glycolysis, and glucose oxidation [43,57,64,65]. Redox homeostasis is also perturbed, leading to elevated levels of reactive oxygen species (ROS) [42,66,67,68,69]. This oxidative stress results from excessive ROS generation by NADPH oxidase, mitochondrial respiration and uncoupled NO synthases, combined with impaired antioxidant defense mechanisms [70].

In addition to structural and metabolic remodeling, diabetes significantly alters cardiac electrophysiology, increasing the risk of arrhythmias. Common abnormalities observed in individuals with diabetes include prolonged QT intervals, increased inter-lead QT dispersion, greater beat-to-beat QT variability, and reduced heart rate variability [71,72,73,74,75,76,77]. These electrical disturbances are thought to arise from altered ion channel expression, autonomic imbalance, and oxidative stress. Epidemiological studies have documented a higher incidence of ventricular tachyarrhythmias, fibrillation and sudden cardiac death among diabetic patients [78,79,80,81,82,83]. A recent study identified sudden cardiac death as the leading cause of mortality in individuals with diabetes under the age of 50, with nearly half of these cases occurring in the absence of underlying coronary heart disease [83].

## 4. Myocyte Ca^2+^ Cycling in Pregnancy and Diabetes

While cardiac adaptation to pregnancy is well documented, there is surprisingly limited information on how pregnancy affects myocyte Ca^2+^ handling. A recent study in mice found that pregnancy has no effect on L-type Ca^2+^ current, Ca^2+^ transient amplitude and kinetics, and SR Ca^2+^ load in atrial myocytes [84]. However, the incidence of spontaneous Ca^2+^ transients was ~twofold higher in cells from pregnant mice, which may contribute to the increased risk of supraventricular arrhythmias observed during pregnancy. Normal Ca^2+^ transients have also been observed in ventricular myocytes from female rats during the immediate postpartum period (<24 h after parturition) [34]. These results align with studies showing that progesterone, at concentrations typical of the third trimester, does not affect Ca^2+^ cycling in cardiac myocytes from female mice [85]. However, progesterone was found to impair contractility by reducing myofilament Ca^2+^ sensitivity [85]. In contrast, estrogen, another hormone elevated during pregnancy, has been shown to reduce both L-type Ca^2+^ current (reviewed in [25]) and systolic [Ca^2+^]_i_ [86]. The discrepancies between the results in myocytes from pregnant animals and myocytes exposed to progesterone/estrogen may reflect differences in hormone concentration, exposure duration, and the complex hormonal milieu of pregnancy. Nevertheless, current evidence suggests that pregnancy does not substantially alter aspects of cardiac Ca^2+^ cycling involved in excitation–contraction coupling. This conclusion is supported by clinical studies reporting no significant changes in cardiac contractility or ejection fraction in pregnant women [19,87,88].

In contrast to the paucity of data in pregnancy, the impact of diabetes on cardiac Ca^2+^ handling has been extensively investigated (recently reviewed in [89,90]). Studies in animal models of both type 1 and type 2 diabetes have consistently found reduced amplitude and slower decay of electrically stimulated Ca^2+^ transients, as well as decreased SR Ca^2+^ load [68,91,92,93,94,95,96,97,98,99]. In rats with late-onset type 2 diabetes caused by expression of human amylin in pancreatic β-cells, Ca^2+^ transient decay is already slowed in the prediabetic stage, while SR Ca^2+^ load remains unchanged [100]. As the disease progresses to overt diabetes, both Ca^2+^ transient amplitude and SR Ca^2+^ content are depressed [68,101]. A longitudinal study in ZSF-1 obese rats found prolonged Ca^2+^ transients at 21 weeks of age, with decreases in Ca^2+^ transient amplitude and SR Ca^2+^ content becoming apparent only at 28 weeks of age [102]. Together, these findings suggest that slowing of Ca^2+^ transient decay is the earliest detectable abnormality in myocyte Ca^2+^ cycling during the development of diabetes.

The reduction in myocyte Ca^2+^ transient and SR Ca^2+^ load during diabetes is primarily due to diminished SERCA activity, resulting from reduced SERCA levels [92,94,96,97,103] and/or elevated expression of its endogenous inhibitor phospholamban [92,98]. There is less agreement regarding the activity of L-type Ca^2+^ channels and NCX in diabetic hearts [92,93,94,95]. Despite the lower SR Ca^2+^ content, several studies (though not all, see [93,94]) have reported an increased frequency of spontaneous Ca^2+^ sparks and elevated SR Ca^2+^ leak in cardiac myocytes from diabetic animals [69,93,95,98,99,101,103,104,105]. This increase in Ca^2+^ leak has been attributed to a higher open probability of RyRs, driven by posttranslational modifications. In particular, enhanced RyR phosphorylation by CaMKII [95,101,104,106] and PKA [101,103,104], as well as increased oxidation [101,105], have been observed in diabetic hearts.

The effect of pregnancy and diabetes on the main components of Ca^2+^ signaling pathways is summarized in Table 1.

## 5. Role of Ca^2+^ Signaling in Cardiac Hypertrophy Associated with Pregnancy and Diabetes

Ca^2+^ plays a key role in the development of cardiac hypertrophy by regulating two major signaling pathways: the calcineurin/NFAT and CaMKII/HDAC pathways. In the first pathway, an increase in [Ca^2+^]_i_ leads to the binding of Ca^2+^ to calmodulin, which activates calcineurin, a Ser/Thr protein phosphatase. Activated calcineurin dephosphorylates members of the NFAT family (NFAT1-4), causing a conformational change that exposes a nuclear localization signal within the regulatory domain of NFAT. As a result, NFAT is translocated from the cytoplasm into the nucleus, where it activates transcription of genes associated with hypertrophic growth [133]. Ca^2+^/calmodulin also activates CaMKII, which phosphorylates class II HDACs (HDAC4 and HDAC5) and causes their export from the nucleus [134]. While in the nucleus, HDAC4 and HDAC5 repress the transcription factor MEF2, thereby inhibiting gene expression. Their export into the cytoplasm relieves this repression, allowing MEF2 to drive the transcription of pro-hypertrophic genes.

The calcineurin/NFAT and CaMKII/HDAC hypertrophy pathways are usually associated with pathological hypertrophy, for example, following pressure overload [3,4]. However, studies in animal models revealed that they are also involved in the cardiac remodeling during pregnancy. In a longitudinal study in mice, Chung et al. [115] found that both the level and activity of calcineurin, as well as NFAT activity (assessed via expression of one of its target genes, modulatory calcineurin-interacting protein 1.4, also known as calcipressin-1), increased during early pregnancy (day 7 of gestation), returned to baseline by mid-pregnancy (day 11), and declined significantly below baseline in late pregnancy (days 18–19). Inhibition of calcineurin blocked the development of cardiac hypertrophy in pregnant mice [121], suggesting that its early activation plays a key role in the cardiac remodeling during pregnancy. Reduced calcineurin/NFAT signaling has also been observed in pregnant rats at gestational days 18–19 [39] and within 24 h after giving birth [34]. This activity returns to the pre-pregnancy level within two months postpartum [34]. However, in female rats with pregnancies complicated by gestational diabetes, the calcineurin/NFAT pathway is reactivated postpartum [34], which may contribute to the increased risk of cardiac hypertrophy and dysfunction observed in females with a history of gestational diabetes. The CaMKII/HDAC signaling is activated in female rats immediately after giving birth and its activity level returns to the baseline within two months post-delivery [34], which suggests that this Ca^2+^-dependent pathway may also contribute to cardiac hypertrophy in pregnancy.

Ca^2+^-dependent signaling also contributes to the development of cardiac hypertrophy in diabetes. Both calcineurin [122,123] and CaMKII [10,101,124,125] are activated in diabetic hearts, in part due to exposure to high glucose levels [10,122,125,135,136]. However, their activation is also evident in prediabetes [100,137,138], when blood glucose is only moderately elevated, indicating that additional glucose-independent mechanisms contribute to their activation. Supporting this, clinical studies have reported a higher incidence of cardiac hypertrophy in individuals with prediabetes [51,52,53]. Activation of CaMKII in diabetes is driven by multiple post-translational modifications, including O-GlcNAcylation (i.e., the O-linked attachment of β-N-acetylglucosamine to Ser/Thr residues), oxidation and S-nitrosylation (reviewed in [139]). In contrast, the mechanisms underlying calcineurin activation are poorly understood. Consistent with increased activity of calcineurin and CaMKII, some studies have reported the nuclear import of NFAT in hearts of rats with both type 1 diabetes [122] and prediabetes [100], as well as the nuclear export of HDAC in prediabetic rats [100]. Despite these findings, the role of the calcineurin/NFAT and CaMKII/HDAC pathways in diabetes-induced cardiac hypertrophy remains incompletely understood.

While calcineurin/NFAT and CaMKII/HDAC hypertrophy pathways (as well as the PI3K/Akt and MAPK/ERK signaling) are activated in both pregnancy and diabetes, the outcomes are divergent, likely due to differences in the duration of pathway activation and the surrounding physiological context. In pregnancy, activation of these pathways is transient, hormonally supported, and accompanied by angiogenesis and pro-survival mechanisms, leading to physiological, reversible hypertrophy. In contrast, diabetes involves chronic activation of hypertrophy signaling, in the presence of hyperglycemia, oxidative stress, and inflammation. Together, these mechanisms drive maladaptive remodeling, fibrosis, and mitochondrial dysfunction, ultimately progressing to heart failure. Thus, the same molecular pathways can mediate either adaptive or maladaptive cardiac remodeling depending on the systemic and cellular environment.

## 6. Role of Ca^2+^ Signaling in the Metabolic Adaptation of the Heart to Pregnancy and Diabetes

As noted in the introduction, Ca^2+^ plays a central role in regulating ATP production and redox homeostasis by activating key rate-limiting enzymes of the Krebs cycle. An increase in mitochondrial Ca^2+^ concentration ([Ca^2+^]_m_) stimulates the Krebs cycle to increase the production of NADH and FADH_2_, which drive the electron transport chain (ETC) and promote ATP synthesis. Enhanced ETC activity also leads to increased ROS generation at complexes I and III. At the same time, elevated [Ca^2+^]_m_ supports the Krebs cycle’s production of intermediates that facilitate NADPH regeneration, helping to counteract the excess ROS production and preserve redox homeostasis. This balance is disrupted under conditions of mitochondrial Ca^2+^ overload, leading to oxidative stress [140]. Conversely, reduced [Ca^2+^]_m_ limits ATP production, impairing the heart’s ability to meet energy demands during periods of high workloads. In parallel, lower [Ca^2+^]_m_ may compromise mitochondrial antioxidant defenses by diminishing NADPH regeneration, leading to oxidative stress.

[Ca^2+^]_m_ is tightly regulated by the balance between Ca^2+^ uptake and efflux. Ca^2+^ uptake occurs primarily through the mitochondrial Ca^2+^ uniporter (MCU) and is driven by the large mitochondrial membrane potential. The channel functions as part of a multi-protein complex that also includes the essential mitochondrial response element (EMRE) and the regulatory subunits mitochondrial calcium uptake 1 and 2 (MICU1/2) [141]. In cardiac mitochondria, the removal of Ca^2+^ from the matrix occurs mainly through the mitochondrial Na^+^/Ca^2+^ exchanger (NCLX), which exchanges one Ca^2+^ ion for 3 Na^+^ ions. Additionally, transient openings of the mitochondrial permeability transition pore also contribute to Ca^2+^ extrusion from the mitochondrial matrix [141].

**Figure 2:**
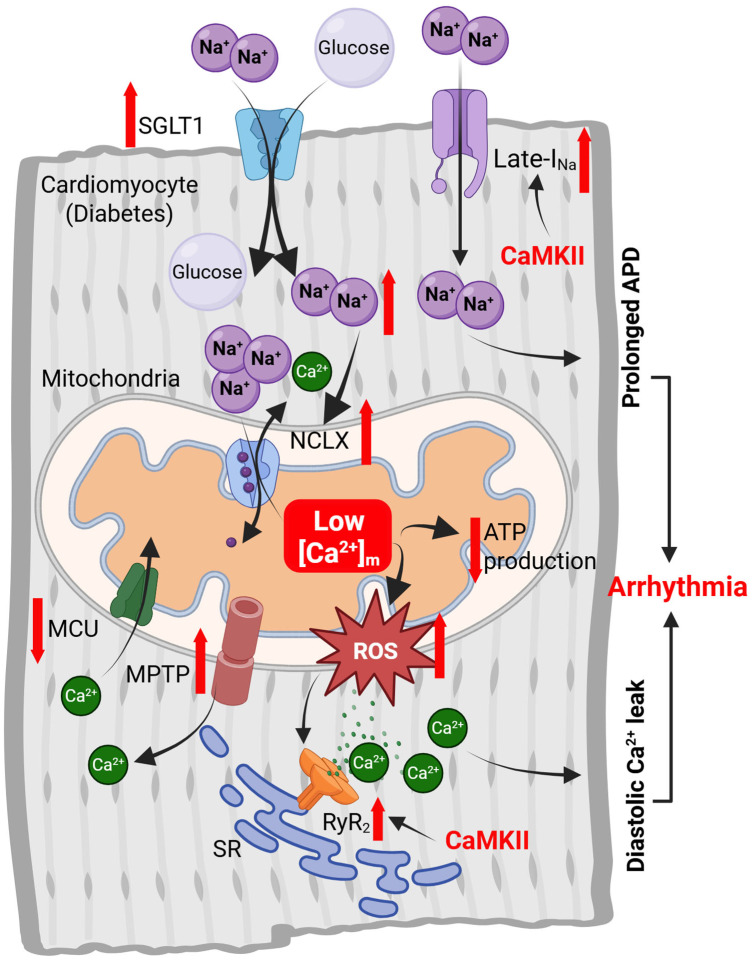
Role of altered mitochondrial Ca^2+^ handling in the metabolic and electrical remodeling of the heart in diabetes. Reduced MCU expression and diminished cytosolic Ca^2+^ transients lead to decreased mitochondrial Ca^2+^ uptake. Conversely, mitochondrial Ca^2+^ efflux is enhanced, driven by upregulated expression of NCLX, its activation by elevated [Na^+^]_i_, and increased sensitivity of MPTP. The resulting decline in [Ca^2+^]_m_ impairs ATP production and promotes oxidative stress. This oxidative stress, in turn, activates CaMKII through oxidation, leading to hyperphosphorylation of RyRs and voltage-gated Na^+^ channels. These modifications increase the SR Ca^2+^ leak and late Na^+^ current, respectively, both of which are recognized contributors to diabetes-associated arrhythmogenesis. Red solid arrows indicate changes in level/expression/activity. SGLT-1: Na^+^-Glucose linked transporter; [Na^+^]_i_: Intracellular Na^+^ concentration; NCLX: Mitochondrial Na^+^/Ca^2+^ exchanger; MCU: Mitochondrial Ca^2+^ uniporter; MPTP: Mitochondrial permeability transition pore; ROS: Reactive oxygen species; CaMKII: Ca^2+^-Calmodulin dependent protein kinase-II; RyR_2_: Ryanodine Receptor-2; APD: Action potential duration; SR: Sarcoplasmic reticulum. Created in https://BioRender.com. (accessed on 2 September 2025).

To date, the effects of pregnancy on cardiac mitochondrial Ca^2+^ transport remain largely unexamined, aside from a single study showing increased sensitivity to mitochondrial permeability transition in hearts from late-pregnant rats [131]. In contrast, several studies have reported that [Ca^2+^]ₘ is decreased in cardiac myocytes from diabetic hearts [69,126,127,128]. The lower [Ca^2+^]_m_ has been attributed to both impaired mitochondrial Ca^2+^ uptake through MCU [69,126,127,128,129,130] and enhanced mitochondrial Ca^2+^ extrusion through NCLX [69] (Figure 2). Consistent with these observations, the expression of MCU [126,127], MICU1 [128] and EMRE [127] is downregulated in diabetic hearts, whereas MCUB [127], a negative regulator of MCU activity, and NCLX [69] are upregulated. Additionally, mitochondria isolated from diabetic rat hearts exhibit increased sensitivity to mitochondrial permeability transition, which further limits [Ca^2+^]_m_ [132]. Beyond transcriptional changes, the activity of MCU and NCLX is controlled by cytosolic Ca^2+^ and Na^+^ levels. As discussed above, cytosolic Ca^2+^ transients remain largely unaltered during pregnancy. However, they are significantly reduced in myocytes from diabetic hearts, further compromising mitochondrial Ca^2+^ uptake. Cytosolic Na^+^ concentration ([Na^+^]_i_) is elevated in both pregnancy [142] and diabetes [143,144,145]. In pregnancy, the increase in [Na^+^]_i_ is primarily due to decreased expression and activity of the Na^+^/K^+^-ATPase [142], while in diabetes, it is mainly driven by excessive Na^+^ influx through the Na^+^-glucose cotransporter 1 [143,144] and, potentially, late Na^+^ current (see below). Elevated [Na^+^]_i_ enhances mitochondrial Ca^2+^ extrusion by increasing the driving force for NCLX, thereby reducing [Ca^2+^]_m_ (Figure 2). Based on these mechanisms, it is plausible to hypothesize that cardiac mitochondrial Ca^2+^ levels may be slightly reduced during pregnancy, despite the lack of direct evidence.

Restoring mitochondrial Ca^2+^ uptake by overexpressing MCU [127] or MICU1 [128] has been shown to improve mitochondrial Ca^2+^ handling and cardiac function in diabetic mice. Similarly, NCLX inhibition with CGP-37157 reduced oxidative stress in hearts from diabetic rats [69]. These findings suggest that decreased [Ca^2+^]_m_ contributes to cardiac oxidative stress and dysfunction in diabetes (Figure 2). Interestingly, while NCLX inhibition significantly reduced spontaneous SR Ca^2+^ release and arrhythmias in diabetic rats, pharmacological activation of MCU, despite producing a comparable increase in [Ca^2+^]_m_, did not yield the same protective effects [69]. In another study, conditional, cardiac-specific deletion of MCU protected mice from mitochondrial dysfunction and arrhythmias induced by high-fat diet [146]. Collectively, these findings indicate that not only the absolute levels of [Ca^2+^]_m_, but also the temporal dynamics of mitochondrial Ca^2+^ fluxes, play crucial roles in determining myocyte performance and overall heart function. The differential effects of MCU activation and NCLX inhibition may reflect their distinct influences on cytosolic Ca^2+^ homeostasis and SR-mitochondrial Ca^2+^ signaling.

TMEM65, a mitochondrial inner membrane protein, has been recently identified as a key player in mitochondrial Ca^2+^ extrusion, either as a mitochondrial Na^+^/Ca^2+^ exchanger itself [147] or as a binding partner for NCLX [148]. TMEM65 is highly expressed in the heart and plays dual roles in mitochondrial Ca^2+^ handling and intercalated disk (ICD) integrity. Recent work shows that loss of TMEM65 impairs mitochondrial Na^+^/Ca^2+^ exchange, leading to Ca^2+^ overload and compromised cardiac function [147,148], while earlier studies demonstrated that TMEM65 is essential for proper connexin43 localization, electrical conduction, and ICD structure, with its deficiency causing cardiomyopathy [149]. Although transcriptomic and proteomic analyses of diabetic hearts reveal widespread remodeling of mitochondrial and ICD proteins, no study has directly examined TMEM65 in diabetic cardiomyopathy, leaving its role an open but mechanistically plausible avenue for research given its clear links to mitochondrial dysfunction, conduction abnormalities, and fibrosis.

## 7. Ca^2+^ Signaling and Electrical Remodeling of the Heart in Pregnancy and Diabetes

Ca^2+^ influences the electrical activity of the heart both as a charge carrier through L-type Ca^2+^ channels and NCX, and as a signaling molecule that regulates various ion channels, primarily via calmodulin and CaMKII. An increase in [Ca^2+^]_i_ leads to L-type Ca^2+^ channel inactivation [134], a process mediated by the binding of Ca^2+^-calmodulin to the α1C subunit of the channel [134]. Concurrently, elevated [Ca^2+^]_i_ activates CaMKII, which enhances L-type Ca^2+^ current through a mechanism known as Ca^2+^-dependent facilitation [134]. NCX activity is also regulated by [Ca^2+^]_i_, both through controlling its thermodynamic driving force and via direct allosteric activation [150]. In addition, CaMKII phosphorylates voltage-gated Na^+^ channels, producing complex changes in their gating [10,134]. The most prominent effect is an increase in the late Na^+^ current, a small component of the Na^+^ current that persists into the plateau phase of the action potential (AP) [10,134]. CaMKII also modulates the gating of several K^+^ currents, including the transient outward K^+^ current (I_to_), the slow delayed rectifier K^+^ current (I_Ks_), and the inward rectifier K^+^ current (I_K1_) [10,151]. Chronic CaMKII activation reduces the density of these currents by altering the transcription of various channel subunits [10,151]. Through these mechanisms, Ca^2+^ signaling profoundly affects the shape and duration of the cardiac AP and, consequently, the QT interval. Sustained CaMKII activation tends to prolong AP duration and the QT interval, contributing to an arrhythmogenic substrate. Ca^2+^ signaling further affects cardiac excitability through CaMKII-dependent phosphorylation of RyRs, which increases the diastolic SR Ca^2+^ leak. The excess Ca^2+^ released during diastole is partly removed from the cell by NCX, generating a transient inward current that can produce delayed afterdepolarizations (DADs) or even trigger spontaneous APs [152,153]. If such ectopic activity occurs synchronously in a cluster of neighboring myocytes (size predictions for such cluster range from a few hundred [154] to several hundred thousand cells [155,156]), it may prematurely trigger ventricular excitation. In the presence of an arrhythmogenic substrate, this mechanism can precipitate ventricular tachyarrhythmias.

As noted earlier, QTc prolongation has been reported in pregnant [23,24,25,26] and diabetic [71,72,73,74,75,76,77] individuals. It is thus not surprising that cardiac AP is longer in both pregnancy [20,84] and diabetes [103,105,157,158,159,160,161]. In ventricular and atrial myocytes from pregnant mice, AP prolongation has been attributed to reduced K^+^ currents due to decreased channel expression [20,84]. However, the potential contribution of Ca^2+^ signaling pathways in this context remains unclear. In contrast, AP prolongation in diabetic hearts appears to be primarily driven by a CaMKII-dependent increase in the late Na^+^ current [103,157,158,159,162]. Interestingly, this late Na^+^ current is attenuated by treatment with the SGLT2 inhibitor empagliflozin [103,163], although the precise molecular target of empagliflozin in the heart has not yet been identified.

SR Ca^2+^ leak is increased in both pregnancy (at least in atrial tissue) [84] and diabetes [69,92,95,98,99,101,103,104,105]. In atrial myocytes from pregnant mice, spontaneous SR Ca^2+^ release was sufficiently large to trigger spontaneous Ca^2+^ transients and aftercontractions [84], suggesting the occurrence of DADs, although DADs were not directly measured. In diabetic animal models, several studies have reported an increased propensity for DADs and ectopic activity in both atrial and ventricular myocytes [101,161,164,165,166,167]. Notably, CaMKII inhibition significantly reduced SR Ca^2+^ leak and the incidence of DADs in ventricular myocytes from diabetic animals [101,165]. Furthermore, inhibition of CaMKII suppressed atrial fibrillation in rats fed diabetogenic diets [167]. Thus, Ca^2+^-dependent mechanisms contribute both to the initiation of arrhythmias and to the development of an arrhythmogenic substrate in diabetes. Similar mechanisms may be active during pregnancy, although the overall arrhythmia risk in this condition is generally low. This may reflect the fact that, in pregnancy, the number of myocytes exhibiting DADs rarely reaches the critical threshold required to trigger an ectopic beat.

## 8. Concluding Remarks

Ca^2+^ signaling plays a central role in the metabolic, structural, and electrical remodeling of heart during both pregnancy and diabetes, as illustrated in Figure 1. Despite fundamental differences between pregnancy as a physiological stress and diabetes as a pathological condition, these states share overlapping Ca^2+^-dependent mechanisms supporting cardiac adaptation, including activation of calcineurin/NFAT hypertrophy signaling, AP prolongation, and increased SR Ca^2+^ leak. The role of Ca^2+^ signaling in cardiac remodeling associated with diabetes is generally well characterized, although some knowledge gaps remain. In contrast, the contribution of Ca^2+^ signaling to cardiac adaptation during pregnancy is poorly understood. Notably, regulation of [Ca^2+^]_m_ and its role in meeting the increased energy demands of the maternal heart during pregnancy remain largely unexplored. Furthermore, the involvement of Ca^2+^ signaling in electrical remodeling of the maternal heart is only beginning to be investigated. Addressing these knowledge gaps will be crucial for advancing our understanding of cardiac adaptation in pregnancy and for identifying novel therapeutic targets to improve maternal cardiovascular health in both physiological and pathological contexts.

## Figures and Tables

**Table 1:** Summary of main findings regarding the impact of pregnancy and diabetes on cardiac Ca^2+^ regulation and signaling.

Pathway/Mechanism	Key Components	Adaptive Regulation/Dysregulation During	
		Pregnancy	Diabetes
Excitation–Contraction Coupling	SERCA	↔ mRNA [20,28,84]	↓ protein and activity [94,95,107,108,109,110,111,112]
	Phospholamban	↓ or ↔ mRNA [28,84]	↑ protein [107], ↔ protein [94,95] ↓ protein [110]
	LTCC	↔ mRNA in atria [84]	↓ I_CaL_ [93,113]
	NCX	↔ mRNA in atria [84]	↑ I_NCX_ [93], ↔ protein [92] ↓ protein [107]
	RyR2	↑ mRNA atria [84]	↑ leak [69,93,95,98,99,101,103,104,105] ↑ protein [95], ↔ protein [92] ↓ protein and activity [93]
	[Ca^2+^]_i_ at rest	Not known	↑ [100,114], ↓ [92,115,116]
	[Ca^2+^]_i_ at peak	Not known	↓ [92,93,107], ↔ [117,118]
	Amplitude of Ca^2+^ transient	Not known	↓ [68,93,107], ↔ [117,118,119]
	Rise rate of Ca^2+^ transient	Not known	↓ [107,119,120], ↓ or ↔ [117]
	Decay rate of Ca^2+^ transient	Not known	↑ [119], ↓ [92,107], ↔ [117,118]
Ca^2+^-dependent hypertrophy signaling	Calcineurin	↑ in early pregnancy ↓ in late pregnancy [39,121]	↑ activity [122,123]
	NFAT		↑ nuclear import [100,122]
	CaMKII	↑ activity [34]	↑ protein [95] ↑ activity [10,101,124,125]
	HDAC		↑ nuclear export [100]
Mitochondrial Ca^2+^ handling	MCU	Not known	↓ expression and activity [69,126,127,128,129,130]
	NCLX	Not known	↑ expression and activity [69]
	MPTP	↑ sensitivity in late pregnancy [131]	↑ sensitivity [132]
	[Ca^2+^]_m_	Not known	↓ level [69,126,127,128]

↔ No change, ↑ increased, ↓ decreased.

## Data Availability

Not applicable. No new data were created or analyzed in this study. Data sharing is not applicable to this article.

## Abbreviations

The following abbreviations are used in this manuscript:

APD	Action potential duration
ATP	Adenosine triphosphate
CaMKII	Calcium/calmodulin-dependent protein kinase II
DAD	Delayed after depolarization
EMRE	essential mitochondrial response element
ETC	Electron transport chain
FADH_2_	Flavin adenine dinucleotide
GLUT	Glucose transporter
HDAC	Histone deacetylase
ICD	Intercalated disk
LTCC	L-type Ca^2+^ channels
MCU	Mitochondrial calcium uniporter
MCUB	Mitochondrial calcium uniporter dominant negative subunit-β
MEF2	Myocyte enhancer factor 2
MPTP	Mitochondrial permeability transition pore
MICU1/2	Regulatory subunits mitochondrial calcium uptake 1 and 2
NADH	Nicotinamide adenine dinucleotide (NAD) + Hydrogen (H)
NADPH	Nicotinamide adenine dinucleotide phosphate
NCLX	Na^+^/Li^+^/Ca^2+^ exchanger, mitochondrial Na^+^/Ca^2+^ exchanger
NCX	Sarcolemmal Na^+^/Ca^2+^ exchanger
NFAT	Nuclear factor of activated T cells
NO	Nitric oxide
PGC-1α	Peroxisome proliferator-activated receptor gamma coactivator 1-alpha
PKA	Protein kinase A
QTc	Corrected QT interval
ROS	Reactive oxygen species
RyR	Ryanodine receptors
SERCA	Sarco/endoplasmic reticulum calcium ATPase
SGLT2	Sodium–glucose cotransporter 2
SR	Sarcoplasmic reticulum
ZSF-1	Zucker fatty and spontaneously hypertensive rat
